# Rat Lung Response to PM_2.5_ Exposure under Different Cold Stresses

**DOI:** 10.3390/ijerph111212915

**Published:** 2014-12-12

**Authors:** Bin Luo, Hongxia Shi, Lina Wang, Yanrong Shi, Cheng Wang, Jingli Yang, Yaxiong Wan, Jingping Niu

**Affiliations:** 1 Institute of Occupational Health and Environmental Health, School of Public Health, Lanzhou University, Lanzhou 730000, China; E-Mails: wangln14@lzu.edu.cn (L.W.); shiyr13@lzu.edu.cn (Y.S.); wangchen2013@lzu.edu.cn (C.W.); yangjingli2013@lzu.edu.cn (J.Y.); wanyx@lzu.edu.cn (Y.W.); niujingp@lzu.edu.cn (J.N.); 2 Lanzhou university Second Hospital, Lanzhou 730030, China; E-Mail: shihongxia0@126.com

**Keywords:** fine particulate matter (PM_2.5_), cold stress, inflammatory response, oxidative stress, rat lung

## Abstract

Ambient particulate matters and temperature were reported to have additive effects over the respiratory disease hospital admissions and deaths. The purpose of this study is to discuss the interactive pulmonary toxicities of cold stress and fine particulate matter (PM_2.5_) exposure by estimating inflammation and oxidative stress responses. 48 Wistar male rats, matched by weight and age, were randomly assigned to six groups, which were treated with cold stress alone (0 °C, 10 °C, and 20 °C (Normal control)) and cold stresses plus PM_2.5_ exposures respectively. Cold stress alone groups were intratracheal instillation of 0.25 mL normal saline, while cold stress plus PM_2.5_ exposure groups were intratracheal instillation of 8 mg/0.25 mL PM_2.5_. These procedures were carried out for three times with an interval of 48 hours for each treatment. All rats were sacrificed after 48 hours of the third treatment. The bronchoalveolar lavage fluid (BALF) was collected for analyzing inflammatory cells and cytokines, and lung homogenate MDA was determined for oxidative stress estimation. Results showed higher level of total cell and neutrophil in the BALF of PM_2.5_ exposed groups (*p* < 0.05). Negative relationships between cold stress intensity and the level of tumor necrosis factor alpha (TNF-a), C-reactive protein (CRP) interleukin-6 (IL-6) and interleukin-8 (IL-8) in BALF were indicated in PM_2.5_ exposure groups. Exposure to cold stress alone caused significant increase of inflammatory cytokines and methane dicarboxylic aldehyde (MDA) and decline of superoxide dismutase (SOD) and glutathione peroxidase (GSH-Px) activity only in 0 °C exposure group (*p* < 0.05). The two-way ANOVA found significant interactive effects between PM_2.5_ exposure and cold stress in the level of neutrophil, IL-6 and IL-8 and SOD activity (*p* < 0.05). These data demonstrated that inflammation and oxidative stress involved in the additive effect of PM_2.5_ exposure and cold stress on pulmonary toxicity, providing explanation for epidemiological studies on the health effect of ambient PM_2.5_ and cold stress.

## 1. Introduction

Temperature is one of the most common physical properties in the natural world. Suitable air temperature is critical for the body to sustain health, while abnormal ones, cold or hot, are harmful. Increasing evidences from epidemiological studies have revealed that lower temperature linked to the increase of adverse health effect, including increased hospital admission and death, exasperation of chronic lung diseases (e.g., chronic obstructive pulmonary disease, COPD). Research reported higher number of inflammatory cells in the lungs of healthy subjects after cold air inhalation [1]. Moreover, animal studies have shown that there were pulmonary cilium destruction, lung inflammation and even airway hyper-responsiveness in rats frequently exposed to cold stress [2,3].

As a critical pollutant of air pollution fine particulate matter (PM_2.5_) has been reported closely related to many adverse effects on respiratory system, including the increase of hospital admission and death and exacerbation of chronic pulmonary diseases [4,5,6,7]. With the increase of ambient PM_2.5_ by 10 mg/m^3^, the exasperation rate of COPD for hospital doubled among 204 US urban counties [8]. Compared with coarse particulate matter (PM_10_), PM_2.5_ may cause more adverse effects because of its smaller aerodynamic diameter and bigger particle amount under similar particle concentration [9]. PM_2.5_ can traverse the blood-air barrier of alveolar, then deposit at lung [10]. It causes toxicities on alveolar macrophages and bronchial epithelial cells to induce inflammation in lung, the mechanism in which mainly include producing inflammatory factors and ROS [11,12,13].

Until now, few studies have studied the interactive effect of particulate matter and temperature on pulmonary health [14,15,16,17,18]. The few previous studies have mainly revealed the additive effect of PM_10_ and low temperature on the emergency hospital admission and death of pulmonary diseases [15,16,18]. Experimental studies have unveiled that cold stress could inhibit airway cilium movement and phagocytes migration and phagocytosis [19,20,21]. We could possibly hypothesize that the inhalation of PM_2.5_ during cold stress might induce more serious damage to the lung. Besides, cold stress often comes with high concentration of PM_2.5_, such as during the appearance of cold air and in the winter. Therefore, we conducted a study aiming to evaluate the interactive effect of PM_2.5_ and cold stress on lung toxicity by assessing several pulmonary biomarkers.

## 2. Methods

### 2.1. Animals

**Figure 1:**
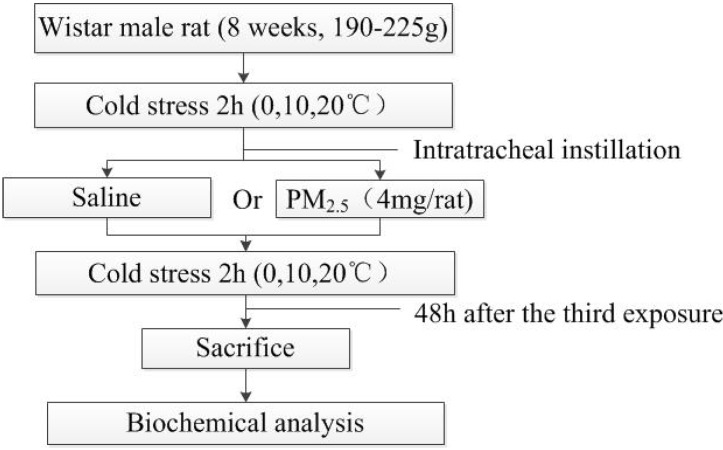
Study design.

The experiments were carried out over eight-week-old Wistar male rats (190–225 g), which were purchased from Gansu University of traditional medicine. Forty-eight rats were assigned to six groups matched with age and weight. Rats were maintained in the animal house in standard cages at 20 °C ± 2 °C, RH 40%–60% and 12:12 h light-dark cycle with free access to laboratory chow and tap water throughout the study. All the animal studies were approved according to the Ethics Committee of Animal Care and Experimentation of the National Institute for Environmental Studies, China. After seven days’ thermal acclimation, all groups were treated according to study design (Figure 1.).

### 2.2. PM_2.5_ Sampling and Processing

Samples of PM_2.5_ were collected between April and May 2014 in Lanzhou, China, were used for the present study. The PM_2.5_ samples were collected onto pre-weighed and pre-dried glass fiber filter, using particulate sampler (TH-150CⅢ, Wuhan Tianhong Instruments Co., Ltd., China) at a flow rate of 100 L/min. Filters were weighed and cut into squares of 1–2 cm^2^, from which particulate matters were removed by agitation for 20 min × 3 times in ultrapure water with ultrasonic shaker. After filtered by six layers of gauze, the solution was centrifuged at 12,000 rpm for 20 min to collect sediment for drying by a vacuum freeze drier (Christ/ALPHA2-4 LD, Germany). The dried PM_2.5_ was kept at −20 °C before diluted for experiment.

### 2.3. Cold Stress and PM_2.5_ Exposure Protocol

Before experiment, PM_2.5_ was diluted in sterile saline in a concentration of 32 mg/mL. Six groups of rats were randomly assigned to different exposure protocols. Protocols were: (1) 20 °C with intratracheal instillation of 0.25 mL sterile saline or PM_2.5_ (8 mg/rat) in animal lab (normal temperature control or PM_2.5_ exposure group); (2) 4 h of 10 °C exposure with intratracheal instillation of 0.25 mL sterile saline or PM_2.5_ (8 mg/rat) in climate simulator; (3) 4 h of 0 °C exposure with intratracheal instillation of 0.25 mL sterile saline or PM_2.5_ (8 mg/rat) in climate simulator. Each protocol was repeated for three times with an interval of 48 h. Therefore, the cumulative dose of PM_2.5_ for each rat is 24 mg. Before instillation of PM_2.5_ or sterile saline, rats were anesthetized through inhalation of diethyl ether.

### 2.4. Bronchoalveolar Lavage

Forty-eight hours after the last intratracheal instillation of sterile saline or PM_2.5_, rats were anesthetized with chloral hydrate (7%, 0.6 mL/100g) and sacrificed through abdominal aorta. Immediately after death, the right lungs were temporarily closed with a haemostatic clamp, and the trachea to the right lung was cannulated. The right lungs were lavaged with 4 × 5 mL volumes of sterile saline. The first lavage fluid was placed in a separate tube for tumor necrosis factor alpha (TNF-α), C-reactive protein (CRP) interleukin-6 (IL-6) and interleukin-8(IL-8) estimations. Tubes were centrifuged at 1500 rpm for 10 min at 4 °C, the supernatant removed and the cell pellet from the first lavage combined with the cells from the same lavage was re-suspended in 1 ml PBS. Total cells were counted directly and neutrophils were counted after stained with Wright-Giemsa dye under Microscope (BX53, Olymbus, Japan). The supernatant of the first lavage fluid was kept at −80 °C for estimation of inflammatory cytokines.

### 2.5. Lung Homogenate

Lung tissue was homogenized in ice-cold saline with glass-homogenizer. The final homogenate concentration was 10%, then centrifuged at 3000 rpm for 10 min at 4 °C to collect supernatant. All supernatant samples were stored at −80 °C for future biochemical analysis.

### 2.6. Biomarker Estimations

Before detection, the supernatants of BALF and lung homogenate stored at −80 °C were recovered at 37 °C in an incubator. The TNF-α, CRP, IL-6 and IL-8 were detected with ELISA kit (RD Biosciences, USA). The contents of total protein and methane dicarboxylic aldehyde (MDA) and the activity of superoxide dismutase (SOD) and glutathione peroxidase (GSH-Px) were determined with assay kit (Nanjing Jiancheng Bioengineering Institute, Jiangsu, China).

### 2.7. Statistics

All data, expressed as mean ± SD, were analyzed using SPSS20.0 IBM statistical software for Windows with a two-way ANOVA (PM_2.5_, cold stress as factors). The differences between paired groups were determined using a paired t-test. The level of *p* < 0.05 was defined as statistical significance.

## 3. Results

### 3.1. Physiological Conditions

**Figure 2:**
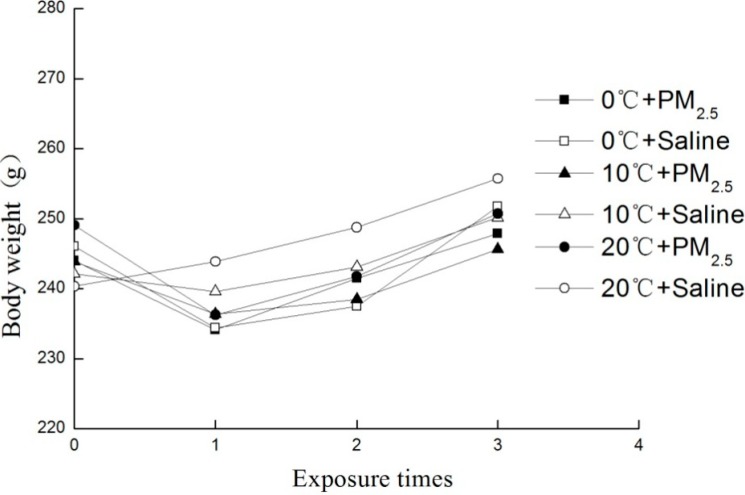
Body weight changes of all groups. The data are presented as mean of each group, n = 8/group.

Among the whole exposure process, no rat died because of exposure factor or any other factors. The Figure 2 showed the body weight changes of all animals through the whole exposure process. Compared with the normal temperature group, the body weight changes of all exposure groups showed a stunted grow trend, especially after the first exposure.

**Table 1:** The total cell numbers and neutrophils in bronchoalveolar lavage fluid (BALF) in all experimental groups.

Cells	0 °C + PM_2.5_	0 °C + Saline	10 °C + PM_2.5_	10 °C + Saline	20 °C + PM_2.5_	20 °C + Saline
Total cell (10^5^)	5.08 ± 1.25 ^a,b^	3.14 ± 0.69 ^b^	3.82 ± 1.09 ^a^	2.79 ± 0.61	3.73 ± 0.93 ^a^	2.33 ± 0.95
Neutrophil (10^5^)	1.76 ± 0.34^a,b^	0.85 ± 0.40	1.28 ± 0.40	0.91 ± 0.43	0.82 ± 0.37	1.01 ± 0.28

Notes: n = 8/group, instillation of PM_2.5_ (8 mg/rat). Data represent as the mean ± SD. Significance: ^a^ *p* < 0.05 compared to the corresponding cold stress alone group; ^b^ *p* < 0.05 compared to the normal temperature group with saline or PM_2.5_.

### 3.2. Cell Counting in Bronchoalveolar Lavage Fluid (BALF)

The Table 1 showed total cells and neutrophils in BALF of different exposure groups. The total cell of all PM_2.5_ exposure groups and the neutrophils of 0 °C plus PM_2.5_ exposure group were higher than their corresponding cold stress alone exposure groups (*p* < 0.05). Compared with normal temperature groups, both 0 °C plus PM_2.5_ exposure group and 0 °C exposure alone group had significantly more total cells, while only 0 °C plus PM_2.5_ exposure group had more neutrophils (*p* < 0.05). Test of between-subjects effects found both cold stress and PM_2.5_ exposure had significant effects over neutrophils (*p* < 0.05), while only PM_2.5_ exposure had significant effects over total cell counts (*p* < 0.05). Cold stress and PM_2.5_ exposure had additive effects on the neutrophils (*p* < 0.05).

### 3.3. Inflammatory Factors in BALF

**Figure 3:**
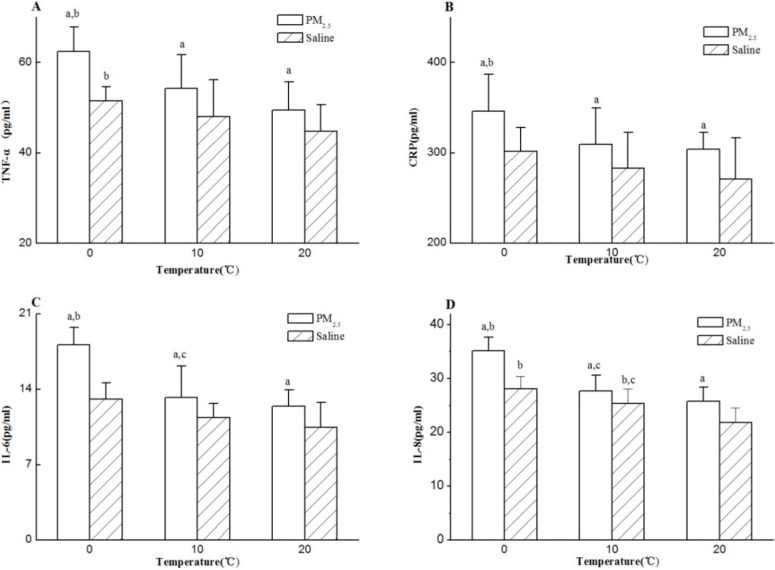
Inflammatory factors level in BALF. (**A**) TNF-ɑ, (**B**) CRP, (**C**) IL-6, (**D**) IL-8. n = 8/group, instillation of PM_2.5_ (8 mg/rat). Data represent as the mean ± SD. Significance: ^a^ *p* < 0.05 compared to the corresponding cold stress alone group; ^b^ *p* < 0.05 compared to the normal temperature group with saline or PM_2.5_; ^c^ *p* < 0.05 compared to 0 °C group with saline or PM_2.5_.

In this study, four inflammatory factors (TNF-ɑ, CRP, IL-6, and IL-8) were determined in the BALF, the results of which were depicted in Figure 3. All PM_2.5_ exposure groups showed significantly higher level of four factors than their corresponding cold stress alone groups (*p* < 0.05). A negative relationship was found between temperature and BALF level of four factors. There were higher levels of IL-8 and TNF-ɑ in the 0 °C exposure alone group compared with the normal temperature group with saline instillation (*p* < 0.05). However, no significant difference was found in other factors among cold stress alone groups (*p* > 0.05). The two-way ANOVAs verified that cold stress and PM_2.5_ had main effects on the contents of TNF-ɑ, CRP, IL-6 and IL-8 (*p* < 0.05), and there were significant interactions only in IL-6 and IL-8 between these two factors (*p* < 0.05).

### 3.4. Pulmonary Oxidant and Antioxidants

**Figure 4:**
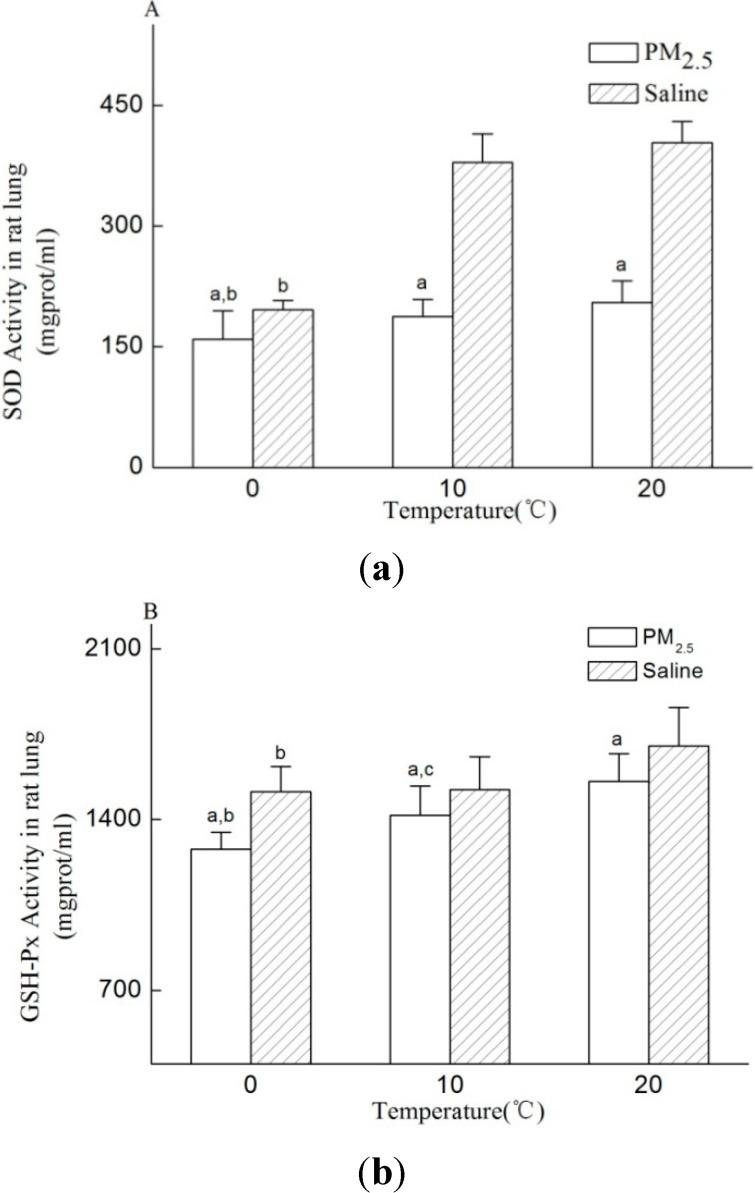

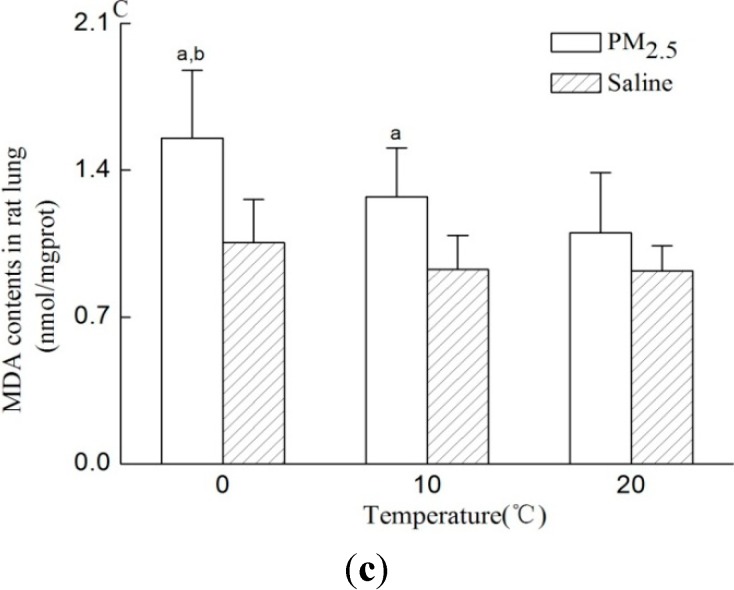
The level of oxidant and antioxidents in rat lung. (**A**) SOD, (**B**) GSH-Px, (**C**) MDA. n = 8/group, instillation of PM_2.5_ (8 mg/rat). Data represent as the mean ± SD. Significance: ^a^ *p* < 0.05 compared to the corresponding cold stress alone group; ^b^ *p* < 0.05 compared to the normal temperature group with saline or PM_2.5_; ^c^ *p* < 0.05 compared to 0 °C group with saline or PM_2.5_.

In order to estimate the effect of cold stress and PM_2.5_ on oxidative injury in rat lung, the levels of SOD, GSH-Px and MDA were detected and showed in Figure 4. The levels of SOD, GSH-Px and MDA in groups with PM_2.5_ instillation were significantly different from their corresponding cold stress exposure alone groups (*p* < 0.05). The activities of both SOD and GSH-Px were found to have a positive relationship with cold stress, which declined with the increase of cold stress intensity, especially among cold stress plus PM_2.5_ exposure groups (*p* < 0.05). The MDA content increased distinctly with the decline of temperature in cold stress plus PM_2.5_ exposure groups. However, no obvious difference was found among the GSH-Px activity and MDA content in cold stress alone groups, except the significantly decline in SOD activity. Through the two-way ANOVAs statistic analysis, the main effects were found both in cold stress and PM_2.5_ exposure in all those three factors (*p* < 0.05), while their interaction only existed in the SOD activity (*p* < 0.05).

## 4. Discussions

Epidemiological studies have unveiled the interactive effects of low temperature and PM over respiratory diseases. The purpose of the present study is to estimate their interactions on lung injury through animal experiment for the first time. In a dust-storm of April 2014 in Lanzhou city, the PM_2.5_ concentration was high as 752 µg/m^3^ and the minimum temperature declined by about 5 °C to 2 °C. As the minute tidal volume of rat is about 0.16 L [22], the PM_2.5_ exposure dose for rat in that day could be about 1.7 mg/d, which is about one fifth of the PM_2.5_ exposure dose (8 mg/rat) used in our study. Concerning the living condition of rat, the cold stress in this study was 0 °C, 10 °C, 20 °C with a scale of 10 °C. Therefore, this study used 8 mg/d of PM_2.5_ exposure and cold stress of 0 °C, 10 °C, and 20 °C as treating factors to estimate the toxicological interactions of PM_2.5_ exposure and cold stress on lung.

Both cold stress and PM_2.5_ exposure were reported to affect the body development by retarding the body weight growth [23,24]. This study showed similar results, each group with cold stress or PM_2.5_ exposure experienced the retarded body weight growth. The total cell influx and TNF-ɑ, CRP, IL-6, and IL-8 in BALF were often used to detect the early inflammatory response in lung [25]. In this study, cold stress alone caused significant increase of total cells, TNF-ɑ and IL-8 in BALF especially in 0 °C group. Additionally, the two-way ANOVAs found its main effect over the four inflammatory factors. These results indicated that cold stress could cause pulmonary inflammation to some extent. Sabnis *et al.* found higher expressions of IL-6 and IL-8 in lung epithelial cells after cold stress of 18 °C for 0–4 h [26]. When combined with PM_2.5_ exposure, a negative relationship was found between the neutrophil counts, TNF-ɑ, CRP, IL-6 and IL-8 and cold stresses. This may suggest that PM_2.5_ exposure added to the effect of cold stress on lung injury. PM_2.5_ exposure alone could induce lung injury represented by the increased level of inflammatory cells and cytokines. The PM_2.5_ exposure of three seasons from European cities induced higher level of TNF-ɑ in BALF of Wistar rat [27]. Wang *et al.* revealed that PM_2.5_ exposure (0.2 mg/rat, 0.8 mg/rat, 3.2 mg/rat) alone increased the total cells, neutrophils, TNF-ɑ and IL-6 in BALF of Wistar rat [24], consistent with the results obtained in this study. When the interactive effect was considered, PM_2.5_ exposure and cold stress had additive effect over the level of neutrophils, IL-6 and IL-8, indicating their additive effect over lung injury. In other words, PM_2.5_ exposure combined cold stress may cause more serious lung inflammation than each of them alone.

It has been suggested that the oxidative stress was one way for ambient particles to affect the lung health [28,29]. The mechanism mainly included attacking pulmonary antioxidant system and promoting the production of ROS in lung, which may be the initiation of inflammation and finally activate apoptosis to induce pulmonary disease [30]. The activity of SOD and GSH-Px and MDA level were often used to estimate the antioxidant system and ROS levels in lung [28,29]. In this study, the activity of SOD and GSH-Px reduced and the MDA level increased after PM_2.5_ exposure, particularly in groups with cold stress. The positive relationship was found between the activity of SOD and GSH-Px and cold stress in PM_2.5_ exposure groups, while a negative relationship existed between MDA level and cold stress. Besides, there were interactive effects between cold stress and PM_2.5_ exposure on SOD activity. These results indicate that PM_2.5_ exposure may increase pulmonary oxidative stress, and cold stress may add to that effect to lead more serious lung injury. However, when cold stress alone was considered, the significant effects over SOD and GSH-Px activity were only found in 0 °C group. Previous studies have not laid focus on the effect of cold stress on oxidative stress, but on the structure alteration of airway and inflammatory cytokines. The present results may suggest us that cold stress could have effect over pulmonary antioxidant system under lower temperature stress. Namely, cold stress may also induce lung injury by increasing oxidative stress in lung.

In this study, the obtained results suggested that leukocyte infiltration, pro-inflammatory cytokines and oxidative stress might involve in the adverse effect of PM_2.5_ exposure on lung. Although the cold stress has affected the level of some pro-inflammatory cytokines and SOD activity in lung to some degree, it may probably play a supplementary role to enhance the pulmonary adverse effect caused by PM_2.5_ exposure. Both PM_2.5_ exposure and cold stress were reported to have adverse effects on lung in both *in vivo* and *in vitro* experimental studies [2,3,24,27,28,30]. Until now, no study has explored the interactive effect of PM_2.5_ exposure and cold stress on respiratory system in animal experimental studies. Salman *et al.* reported that the phagocytic activity of peritoneal macrophage over latex particles was inhibited after cultured at 24 °C for 60 min [31]. A research over human unveiled that cold stress inhibited leukocyte migration and phagocytic activity of phagocytic cells [19,21]. There is possibility that cold stress may also inhibit the phagocytic activity of pulmonary macrophage over PM_2.5_ and enhance the pulmonary toxicity of PM_2.5_. Additionally, experimental study found cilium injury by ciliated epithelial loss after frequently cold stress [19,21]. In a cold environment of 4 °C for 1 h, the constricted pulmonary vascular declined the pulmonary blood volume in rabbit, which inhibited the supply of immune cells in lung and cilium movement [32]. Cilium in respiratory system is the first defense line against PM_2.5_, the injured and retarded cilium would promote the inhalation of PM_2.5_ to the lung. If these assumptions are possible, the pulmonary defense system against PM_2.5_ will be seriously inhibited by cold stress, which may enhance the pulmonary toxicity caused by PM_2.5_ exposure. However, these still need to be analyzed in future studies. Another limitation of our study is that we did not study specific chemical components of PM_2.5_, so we could not identify the causative constituents in the observed adverse effects. Therefore, the correlation between chemical elements and toxic effects should be discussed specifically in the future. Additionally, histological result of the lung was not discussed in this study because of some technique problems, so we could not provide any information about the structure change of lung issue, such as the spaces of alveolar ducts and sacs, the thickening of interstitial connective tissues, inflammatory cell infiltration, *etc*., in treated rats *vs*. control. However, these limitations would be covered in the next step of our project.

## 5. Conclusions

In summary, our results indicated that both cold stress and PM_2.5_ exposure could cause adverse effects on lung and cold stress may enhance the toxic effect of PM_2.5_ exposure. The interactive toxic mechanism for cold stress and PM_2.5_ exposure to induce higher occurrence of respiratory disease events could be through provoking oxidative stress and higher production of pro-inflammatory cytokines.
